# Validating self-reported cervical cancer screening among women leaving jails

**DOI:** 10.1371/journal.pone.0219178

**Published:** 2019-07-01

**Authors:** Shelby Webb, Patricia J. Kelly, Joi Wickliffe, Kevin Ault, Megha Ramaswamy

**Affiliations:** 1 Department of Preventive Medicine and Public Health, University of Kansas School of Medicine, Kansas City, Kansas, United States of America; 2 (Retired) School of Nursing, University of Missouri Kansas City, Kansas City, Missouri, United States of America; 3 Department of Obstetrics and Gynecology, University of Kansas School of Medicine, Kansas City, Kansas, United States of America; Ordu University, TURKEY

## Abstract

**Background:**

Despite women with criminal justice involvement reporting routine Papanicolaou (Pap) testing, significant disparities in cervical cancer outcomes exist when compared to women without criminal justice involvement. A possible reason for the discrepancy is that this group of women may be misreporting Pap testing. The objective of this study was to validate self-reported cervical cancer screening among women leaving jails.

**Methods:**

We used three methods to validate self-reported cervical cancer screening for women recently released from jail: 1) Medical record review; 2) Semi-structured interview; 3) Pap test knowledge survey. After validating women’s self-reported Pap tests with a review of their medical records, we scored interviews for Pap test recall, and used Pap test knowledge survey scores to compare scores between women who accurately reported Pap tests vs. those who did not.

**Results:**

Sixty-one percent (N = 14/23) self-reported cervical cancer screenings were accurate per medical record review. Comparing participants who did and did not accurately self-report a Pap test, we found a significant difference in Pap test recall scores (1.90 vs. 0.00, t = 3.87, p < .01) and Pap test knowledge scores (13.50 vs. 12.13, t = 2.42, p < .05).

**Conclusion:**

Self-report of cervical cancer screening was more likely to be accurate if a woman’s Pap test knowledge was high. Clinicians might take extra care in describing screening and distinguishing between Pap tests and pelvic exams to support the cervical health of women with lower knowledge.

## Introduction

Cervical cancer was once a leading cause of death among women of childbearing age. Over the last 40 years, interventions such as the Papanicolaou (Pap) test for cervical screening, human papillomavirus (HPV) vaccine, and access to health care services through increased insurance coverage have led to the decline of cervical cancer [1]. These interventions have not been effective in addressing justice-involved women’s cervical health needs. This population has higher rates of cervical cancer, higher rates of histories of abnormal Pap tests, and lower rates of Pap testing [2]. The Sexual Health Empowerment (SHE) project for cervical health literacy and cancer prevention was developed to address this disparity as an intervention delivered to jailed women [3,4]. In assessing the long-term effectiveness of this intervention, a helpful metric has been to establish when these women received their last Pap test in post-intervention questionnaires. The United States Preventive Services Task Force recommends women age 21 to 65 get a Pap test every three years [5]. On the SHE one-year post-intervention survey, 82.0% of respondents reported having a Pap test in the last three years [6]. This rate was higher than reported Pap tests in the general population, where, in a survey of 1,926 U.S. women, 73.2% of respondents reported having a Pap test in the last three years [7].

Recall bias can challenge the validity of a participant’s self-reported Pap test. Such recall may present other challenges, as well. A pelvic exam is performed by a healthcare professional to examine reproductive organs. While often including a Pap test, a pelvic exam may be performed for many reasons unrelated to cervical cancer screening. Limited cervical health literacy may lead women to mistakenly report a pelvic exam as a Pap test [7]. Cervical cancer screening awareness among justice-involved women is low, with studies showing that incarcerated women consider any range of women’s health problems as “abnormal Pap test results,” and confusion about the etiology of cervical cancer, screening recommendations, and need for screening [8,9].

Self-reports of medical services are often inadequate and downward adjustment is suggested to correct for overestimation [10–20]. While focused on vulnerable and minority populations, much of this research is over a decade old and few studies focus on the group of women who move frequently between jails and communities. The goal of this project was to address that gap in the literature by validating self-reported cancer screening Pap tests among women recently released from jail.

## Materials and methods

### Study design

We used a validation study research model, which is most commonly a comparison of patient self-report with medical record review for a given procedure, with most studies suggesting that there are limitations to self-report alone, based on patient recall and knowledge. Thus, in order to complete this validation study, *four* sources of data were compared, to include not only patient self-report and medical record review, but also assessments of patient recall and knowledge. The *first* source was the self-report of a last Pap test from participants’ post-intervention follow-up surveys at one, two, and three years post-intervention. The *second* source was the medical records of participants who both self-reported a Pap test post-intervention and gave consent for the release of their medical records to the study team. To understand more about how and why self-reporting does not always match medical records, a *tertiary* source was collected: semi-structured interviews asking about a participants’ last Pap test. The *fourth* data source was a participant’s Pap test knowledge score, a composite score from 15 true or false questions on the same annual survey that a participant self-reported a Pap test.

### Sample

There were 182 SHE intervention completers in the parent study [4]. This total excluded three participants who had completed the intervention, yet were flagged for various reasons, including participation in the pilot intervention or not completing the intervention in the assigned intervention group. For the purposes of this study, flagged participants were included in eligibility screening because the focus was on verification of self-reported Pap tests, rather than on intervention outcomes.

Therefore, participants were selected from 185 SHE invention completers who reported a Pap test post-intervention in the one, two, or three-year follow-up surveys. Of the 96 participants that met these criteria, 23 participants signed a release of medical information and 16 participated in a semi-structured interview (Figure in S1 Fig). All participants were between the ages of 30 and 58 and formerly incarcerated in the Kansas City metropolitan area.

### Procedures

For each of the 96 eligible participants, a minimum of two contact attempts were made to schedule an interview and collect an authorization of medical record release. Some contact numbers were disconnected, unable to accept calls, or had full voicemail inboxes. Some participants were contacted through Facebook. If a participant did not respond to any contact attempts they were categorized as “could not be contacted.” Participants were also excluded if they had moved outside the Kansas City metropolitan area or were presently incarcerated. Incarcerated participants were excluded due to difficulties in accessing them within reasonable time frames. All participants were informed that medical record release was completely voluntary and would not affect their participation in the rest of the study. A $10 incentive was given to those who participated in an interview to compensate for time. Interviews took place in fast food restaurants and participants’ homes. The interviews were transcribed modified verbatim by the first author after all in-person interviews had been completed.

The study protocol was approved by the University of Kansas Medical Center Institutional Review Board.

### Data

*Self-report of Pap tests* was taken from researcher-administered follow-up surveys from the original SHE intervention study. Participants who self-reported an up-to-date Pap test were identified by reviewing follow-up surveys for each participant and checking two questions: 1) Did you have a Pap test in the last year? and 2) When was your last Pap test? The second question allowed participants to identify how many years ago their last Pap test was, so was an approximation and not an exact date. If the answer to the first question was “Yes” or if the answer to the second question was within the last three years, the participant was considered to have an up-to-date Pap test.

To obtain *medical records to check for accuracy of self-report*, participants who self-reported an up-to-date Pap test were contacted at their last known contact number to see if they would sign a release of medical information. Signed releases were faxed to the medical institution identified by participants as the place they got their last Pap test. Participants’ self-reporting of having a Pap test was compared to medical records. Notations of “Pap” or “Cytology—Pap” in the medical records, within two years of the self-reported Pap test, were considered to have had a Pap test. This two year buffer was used to account for difficulties in recalling the exact year the Pap test took place.

*Semi-structured interviews for Pap test recall* were conducted to distinguish whether participants had a pelvic exam rather than a Pap test (text in S1 Text). Questions also elicited participants’ experiences during a Pap test or gynecological visit, including their interactions with their health care provider and how their results and need for follow-up were communicated.

*Pap test knowledge* was a composite score from 15 true or false questions from the annual survey on which a participant self-reported a Pap test (text in S2 Text). This scale was developed for an earlier intervention targeting low-income Hispanic women and has been used throughout the original SHE intervention [21]. If participants reported up-to-date Pap tests on two or more follow-up surveys, average scores were computed. Scoring was from 0–15. The scale had an alpha of .88, suggesting it is a reliable assessment of a participant’s Pap test knowledge.

### Data analysis

Semi-structured interviews were audiotaped and transcribed. Using content analysis, two researchers used an iterative process to score transcriptions for Pap test recall. Areas of disagreement were discussed until consensus was reached. Four topic areas indicated that a Pap test was done: cytobrush use, the purpose of a Pap test, results specific to a Pap test, and follow-up specific to a Pap test. Participants were given a score of 1 or 0 in each domain for a total score of 0–4.

Bivariate chi-square analyses were used to assess significant differences between validation study participants and parent study participants. Non-parametric Wilcoxon-Mann-Whitney tests were conducted to compare differences in semi-structured interview scores and Pap test knowledge scores between participants who did and did not accurately self-report Pap tests. These test were performed using SAS Studio software 3.7 (SAS Institute Inc., Cary, North Carolina).

## Results

Participants in the parent SHE study and those in the validation study were compared based on sociodemographic characteristics. At baseline, participants in the validation study (N = 23) were similar with no statistically significant differences on employment prior to incarceration, homelessness, insurance status, having a primary care doctor, having a medical home, or having an up-to-date self-reported Pap test, compared to the rest of the participants in the parent study (N = 162). Participants in the validation study were more likely to be Black (*p*<0.05) and more likely to have completed a high school education or more (*p*<0.05) compared to the rest of the parent study participants.

Of the 23 participants included in this validation study, the average age was 40.1.

Participant characteristics are described in Table 1.

**Table 1:** Participant characteristics, N = 23.

	Mean	SD
**Age**	40.1	9.71
	**N**	**%**
**Race/Ethnicity**		
White	7	30.4
Black	13	56.5
Other	3	13.0
**High school education or higher**	18	78.3
**Employed prior to incarceration**	7	30.4
**Homeless at time of arrest**	2	8.7
**Insured prior to incarceration**	12	52.2
**Has primary care doctor**	20	87.0
**Has medical home**	13	56.5

Medical records were obtained for all 23 participants that signed a release of medical information. In these medical records, there were 14 (60.9%) confirmed Pap tests. Nine (39.1%) records either had no mention of Pap tests or had Pap tests that were significantly older than those self-reported. The results of the medical record retrieval and the interviews are presented in Table 2.

**Table 2:** Participant data validation.

Participant	Medical Record	Pap Test Knowledge Average (0–15)^a^	Interview Score (0–4)^b^
P1	Pap	14	1
P2	No Pap	14	0
P3	Pap	14	1
P4	No Pap	12	NA
P5	Pap	14	NA
P6	No Pap	9	NA
P7	Pap	14	NA
P8	No Pap	12	NA
P9	No Pap	13	NA
P10	No Pap	10	0
P11	No Pap	13	0
P12	Pap	14	1
P13	Pap	14	1
P14	Pap	13	3
P15	Pap	12	2
P16	Pap	11	3
P17	No Pap	13	NA
P18	Pap	13	1
P19	Pap	14	2
P20	Pap	14	3
P21	Pap	15	1
P22	No Pap	14	0
P23	Pap	13	2

^a^ Pap test knowledge is the composite score of 15 true/false questions on annual surveys. In cases where a participant self-reported a Pap test on more than one survey, the average of Pap test knowledge scores was taken.

^b^ Interviews were scored from 0–4 on 4 domains: cytobrush use, mention of cervical cancer/HPV, results specific to a Pap test, and follow-up specific to a Pap test.

NA—Participant did not complete an interview.

Semi-structured interviews were completed with the 16 of the participants that could be reached and consented to an interview. The interviews took an average of five minutes. Comparisons of Pap test knowledge and interview scores between each group (those that accurately self-reported a Pap test and those that did not accurately self-report a Pap test) are presented in Table 2. The average interview score was 1.75 for a participant who accurately self-reported a Pap test and 0.00 for a participant who did not accurately self-report a Pap test (z = -2.97, *p*<0.01). The average Pap test knowledge score was 13.50 for a participant who accurately self-reported a Pap test and 12.22 for a participant who did not accurately self-report a Pap test (z = -2.06, *p* = 0.04).

While many participants scored high on their semi-structured interview, recalling a Pap test was challenging for the four that did not accurately self-report one. These four all received a score of 0 for their interview content analysis. One belief that led to the incorrect self-report by each of these participants was the idea that if a speculum was used, they had received a Pap test. The medical records from three incorrect self-report participants—two who were interviewed and one who was not—showed that they did have various pelvic-related care: two had an STI screening and one had urology care. These participants did not understand the difference between a Pap test and other types of gynecological medical care or screenings.

## Discussion

These findings show that the accuracy of a cervical cancer screening self-report is related to knowledge of the procedure. Overall, 60.9% of the 23 medical records confirmed accurate self-report of Pap tests. This is lower than the 85.6% validation found between self-report and administrative records of adults recently released from prison in another study [22]. However, that study focused on the broad categories of primary care, emergency department use, hospitalization, and prescription drug use [22]. The agreement of the present study was in line with other existing literature on validation of Pap tests [10–13,15].

Those who inaccurately self-reported a Pap test equated pelvic exams with receipt of a Pap test. The findings of this study are consistent with another study of incarcerated women in the Kansas City area that found Pap tests were often thought of as an all-inclusive screening for gynecological and pelvic health issues. Pelvic exam findings such as abnormal bleeding, STIs, bacterial vaginosis, yeast infections, and ovarian cysts were considered “abnormal Pap test results” by many of the women in interviews [8].

Pap test knowledge was associated with accurate Pap test self-report. Even with adequate overall health literacy, cervical health knowledge can vary. Although one study reported that 91.1% of incarcerated women had an adequate health literacy score, many were not able to accurately describe the purpose of the Pap test when probed qualitatively [9]. In that study, while some did correctly identify a Pap test as a procedure to screen for cervical cancer, they also incorrectly believed the Pap test was used to check for STIs or to initiate birth control.

The primary limitation of our validation study was the small sample size. Signing a release of medical information was not required to be part of the study. Had these releases been routinely collected as participants completed the intervention, more robust data on the accuracy of self-reports would have been possible. Women leaving jails often have inconsistent contact information, which can make follow-up and further data collection difficult. This reality may have resulted in a low response rate when medical record releases and interviews were requested. Another limitation is that medical records were only collected from the institution that a participant identified as the place she got her last Pap test. If a participant received an up-to-date Pap test at a location she did not identify, this could not be validated.

This study supports interventions that aim to increase cervical health knowledge in vulnerable populations as a method to increase cancer screenings. If Pap test knowledge can be increased, the likelihood of correctly self-reporting a Pap test is higher. Increased recall of a Pap test may in turn result in better adherence to recommended life-long screening, reducing the disparity of cervical cancer in incarcerated women and other vulnerable populations.

In conclusion, the self-reporting of cervical cancer screening is more likely to be accurate in justice-involved women if a person’s knowledge of a Pap tests and Pap test procedures are high. Pelvic exams and STI tests are often mistaken for Pap tests when knowledge of a Pap test is low. Clinicians may work harder to provide accurate information and assess knowledge of procedures during gynecological exams, in order to support the cervical health of vulnerable women.

## Supporting information

S1 Fig Flow chart for selection of study participants. (DOCX)

S1 Text Prompts for semi-structured interviews. (DOCX)

S2 Text Survey items for Pap knowledge scale. (DOCX)
