# Supplementary material for: Validating self-reported cervical cancer screening among women leaving jails
Source: PLoS One. 2019 Jul 1;14(7):e0219178. doi: 10.1371/journal.pone.0219178 (PMC6602293; doi:10.1371/journal.pone.0219178)
Supplement: S1 Fig — (DOCX) [file pone.0219178.s001.docx]

**S1 Fig. Flow chart for selection of study participants**

Eligible participants were SHE intervention completers (N=185).

­

Participants excluded for not self-reporting a Pap on an annual survey (n=89)

Participants signed medical release (n=23)

In-depth Pap interviews completed (n=16)

Medical records received (n=23)

Participants excluded (n=73)

- Could not be contacted (n=39)
- Moved (n=10)
- Incarcerated (n=9)
- Declined (n=3)
- Lost interest or failed to reschedule (n=12)

Participants that self-reported a Pap post-intervention on their 1, 2, or 3 year survey (n=96)
