# Supplementary material for: Validating self-reported cervical cancer screening among women leaving jails
Source: PLoS One. 2019 Jul 1;14(7):e0219178. doi: 10.1371/journal.pone.0219178 (PMC6602293; doi:10.1371/journal.pone.0219178)

S2 Text. Prompts for Semi-Structured Interviews

1. Where did you get your last Pap?
   1. What type of health facility was it? (i.e. Hospital, family medicine practice, clinic, health department, emergency department)
   2. Will you get your next Pap at this same location?
2. Did your healthcare provider explain the tools they used during your last Pap? *(Show pictures of tools, see next page)*
   1. Was a speculum used?
   2. Was a cotton tipped applicator used?
   3. Was a cytobrush used?
   4. Was a gynecological spatula used?
3. Did your healthcare provider talk to you about the Pap before, during, or immediately after the exam but during the same visit?
   1. Can you describe what your provider discussed with you?
   2. Did you ask your provider questions during the visit?
      1. Can you give me an example of one of the questions you asked your provider?
      2. Did you understand the answers your provider gave you?
4. Did your healthcare provider call you back to describe the results of your last Pap?
   1. What were the results of your last Pap?
      1. *(If normal)* Did your healthcare provider say when you should schedule your next Pap smear?
      2. *(If abnormal)* Did your provider schedule a follow-up visit or suggest an action plan?

Plastic and metal speculum


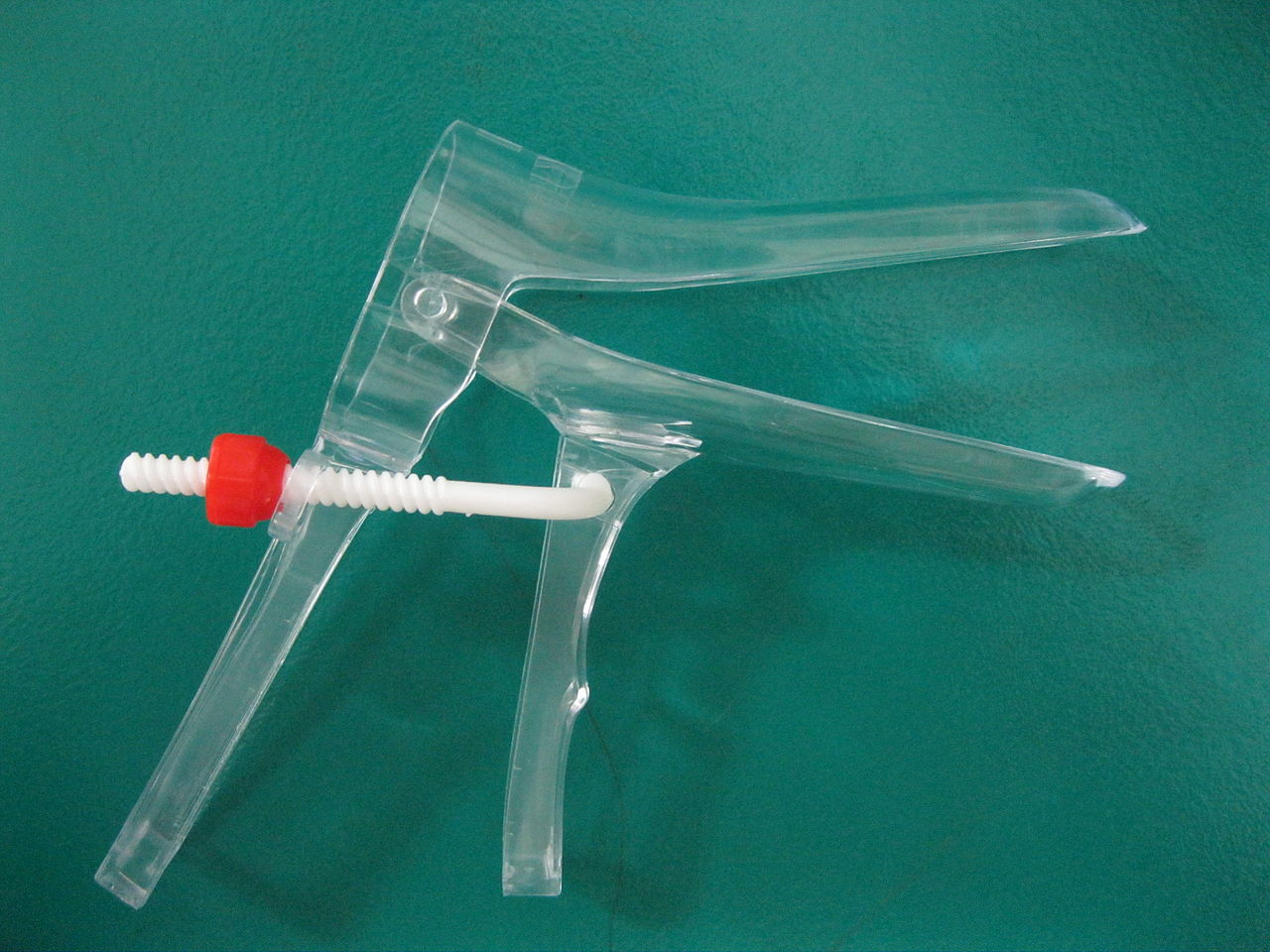

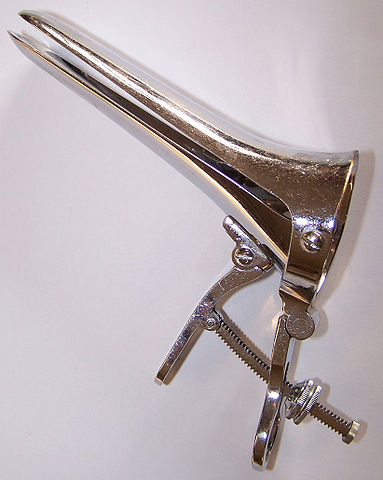


Cotton tip applicator


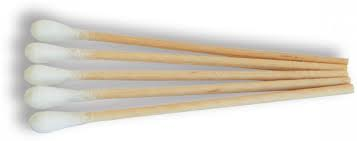


Cytobrush


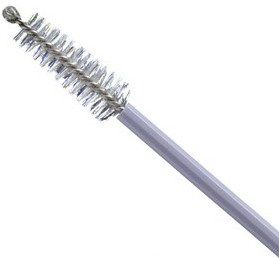


Gynecological spatula


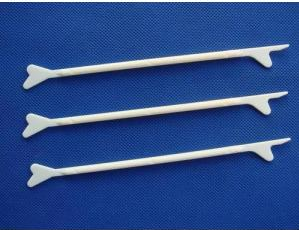

Supplement: S1 Text — (DOCX) [file pone.0219178.s002.docx]
