# Supplementary material for: Validating self-reported cervical cancer screening among women leaving jails
Source: PLoS One. 2019 Jul 1;14(7):e0219178. doi: 10.1371/journal.pone.0219178 (PMC6602293; doi:10.1371/journal.pone.0219178)
Supplement: S2 Text — (DOCX) [file pone.0219178.s003.docx]

S3 Text. Pap Test Knowledge Survey Items

from: Fernández ME, Gonzales A, Tortolero-Luna G, Williams J, Saavedra-Embesi M, Chan W, & Vernon SW. Effectiveness of Cultivado La Salud: A breast and cervical cancer screening promotion program for low-income Hispanic women. Am J Public Health. 2009; 99(5):936-943. doi:10.2105/AJPH.2008.136713

Answer options: True, False, Don’t know/prefer not to answer

1. Human Papillomavirus (HPV) can lead to cervical cancer.

2. A lack of hygiene can cause cervical cancer.

3. People having sex without condoms have higher risk of getting cervical cancer.

4. Pap screening is done through drawing your blood.

5. Women who have gone through menopause do not need to be screened for cervical cancer/have Pap screenings.

6. If you have cervical cancer you would feel pain.

7. Women who do not have regular cervical cancer screenings (Pap tests) are more likely to have advanced cervical cancer when they were diagnosed.

8. After women stop having children, they do not need to have screenings for cervical cancer (Pap tests).

9. Screening for cervical cancer (getting Pap tests) is not important for a woman under 50 years.

10. Only women who have had many sex partners need to get screened for cervical cancer/have a Pap test.

11. Screening for cervical cancer/Pap tests can only detect advanced (invasive) cervical cancer.

12. Screening for cervical cancer (Pap test) is necessary even if there is no family history of cancer.

13. Once I have a screening for cervical cancer (Pap test) that shows no problems, I don’t need to have any more.

14. I need to be screened for cervical cancer (have a Pap test) only when I experience problems like pain or vaginal bleeding that is not my period.

15. Screening for cervical cancer (Pap tests) can detect problems before they become cancer.
